# Rapid test to detect insecticide resistance in field populations of *Spodoptera frugiperda* (Lepidoptera: *Noctuidae*)

**DOI:** 10.3389/fphys.2023.1254765

**Published:** 2023-08-23

**Authors:** Kai-Kai Mao, Hong-Ran Li, Jing-Yun Zhu, Ming-Hui Jin, Peng Wang, Yan Peng, Yu-Tao Xiao

**Affiliations:** Agricultural Genomics Institute at Shenzhen, Chinese Academy of Agricultural Sciences, Shenzhen, China

**Keywords:** *Spodoptera frugiperda*, insecticide, resistance monitoring, diagnostic kit, accuracy verification

## Abstract

**Introduction:** The development of insecticide resistance in *Spodoptera frugiperda* populations is a serious threat to the crop industry. Given the spread of invasive resistant populations, prospective monitoring should be accelerated, and the development of diagnostic tools for rapid and accurate assessments of insecticide resistance is essential.

**Methods:** First, the discriminating dose and diagnostic time of the kit were determined by the glass vial method based on a susceptible strain. Then, pests that were collected from field populations were used to determine their susceptibility to seven insecticides by using the diagnostic kit. Finally, the accuracy of the kit was verified based on correlation analyses and the likelihood of insecticide control failure was assessed.

**Results:** Here, we describe a diagnostic kit that enables the rapid detection of resistance to chlorpyrifos, bifenthrin, deltamethrin, lambda-cyhalothrin, phoxim, chlorantraniliprole and chlorfenapyr within 1-2 h in *S. frugiperda* at diagnostic doses of 0.98, 0.84, 0.38, 1.64, 0.0082, 1.75 and 0.65 μg/cm^2^, respectively. The linear equation between mortalities under diagnostic doses and actual resistance ratios measured by the diet-overlay bioassay was determined. The high correlation indicates that the insecticide resistance levels diagnosed by the kit were consistent with the results of the diet-overlay bioassay. Moreover, we found a significant negative correlation between diagnostic mortality and the likelihood of control failure for bifenthrin (*r* = −0.899, *p* = 0.001), deltamethrin (*r* = −0.737, *p* = 0.024) and lambda-cyhalothrin (*r* = −0.871, *p* = 0.002).

**Discussion:** The insecticide resistance diagnostic kit for *S. frugiperda* is a user-friendly tool (portable, short detection time). Its excellent performance qualifies the kit as a reliable screening tool for identifying effective insecticides in sustainable resistance management.

## 1 Introduction

The fall army worm (FAW), *Spodoptera frugiperda* (Lepidoptera: *Noctuidae*), is a major transboundary migratory pest that is native to tropical and subtropical regions of the Americas (Xiao, 2021). Currently, its presence has rapidly expanded to more than 100 countries worldwide (Blanco et al., 2016; Burtet et al., 2017; Ganiger et al., 2018; Guo et al., 2022). It invaded China through the Yunnan Province on 11 December 2018 and quickly spread across the country via its long-distance migration abilities (Sun et al., 2021). *S. frugiperda* is a highly polyphagous pest that attacks more than 353 host plants, causing major damage to maize, sugarcane, rice, wheat, sorghum, other vegetable crops and cotton. This represents a great threat to food security and results in economic loss (Baudron et al., 2019; Xiao, 2021).

Although maize hybrids that express *Bacillus thuringiensis* insecticidal proteins as well as biological control agents have been developed and applied against *S. frugiperda* (Jin et al., 2021a), chemical control remains the most common practice for the management of *S. frugiperda* worldwide (Omoto et al., 2016; Muraro et al., 2021). However, as a consequence of the pest’s genetic plasticity, high fecundity and particularly strong selection pressure, *S. frugiperda* has developed resistance against 45 active pesticide ingredients, and its reported cases of resistance amount to 200 (APRD, 2023). In particular, considering that the invaded *S. frugiperda* populations in China have a genetic background of multi-insecticide resistance due to its origin in the tropical and subtropical regions of Americas (Li et al., 2020a; Zhao et al., 2020), subsequent use of higher concentrations of these insecticides to maintain control will necessarily result in an increased frequency of resistant individuals and eventually a control failure (Zhang et al., 2016; Li et al., 2020b). The primary strategy for mitigating the detrimental effects of insecticide resistance is the development of an insecticide resistance management plan, in which the concept of resistance monitoring was developed as a means to minimize overuse and prevent or at least delay the development of resistance (Ruscoe, 1987; Mao et al., 2019a; Liao et al., 2020). In *S. frugiperda*, current resistance monitoring methods are mainly based on the diet-overlay bioassay or leaf disk method (Lira et al., 2020; Chen and Palli, 2022). Although these methods can be used effectively to monitor resistance to insecticides, one of their limitations is their complexity, which requires a range of operational procedures and specialist technicians, and that it takes at least 48 h to obtain results. This is contrary to the interests of growers, who need reliable data in a short amount of time, especially during periods of high pest infestation. Therefore, a reliable and rapid method of resistance detection needs to be developed and correlated with the field efficacy of the corresponding insecticide, thus scientifically avoiding the use of ineffective insecticides and contributing to reversing existing levels of resistance.

The glass vial bioassay, as a user-friendly method (short detection time and easy to perform), has been successfully used to detect insecticide resistance levels in a wide range of insects, such as *Drosophila suzukii*, *Tetranychus urticae*, *Phlebotomus papatasi* and *Lutzomyia longipalpis* (Kwon et al., 2010; Denlinger et al., 2016; Van Timmeren et al., 2019). Previous research has developed a diagnostic kit for the rapid detection of resistance to imidacloprid, nitenpyram, clothianidin, dinotefuran, thiamethoxam, isoprocarb and chlorpyrifos in *Sogatella furcifera* based on the glass vial bioassay (Mao et al., 2021). Huang et al. (2022) developed an insecticide phenotypic resistance diagnostic kit for *Aphis gossypii*, where the results can be obtained within 3 h. These successful applications show that an efficient and rapid method of detecting the resistance levels of *S. frugiperda* to insecticides will be possible.

Therefore, the objective of this study was to develop a diagnostic kit that provides the estimation of resistance to chlorpyrifos, bifenthrin, deltamethrin, lambda-cyhalothrin, phoxim, chlorantraniliprole and chlorfenapyr in 1–2 h. Multiple field populations of *S. frugiperda* will be used to verify the accuracy and efficiency of this diagnostic kit. The application of this kit can help practitioners or farmers quickly screen available agents and filter out ineffective insecticides, providing an important technical tool for pest resistance management.

## 2 Materials and methods

### 2.1 Insect collection

**FIGURE 1:**
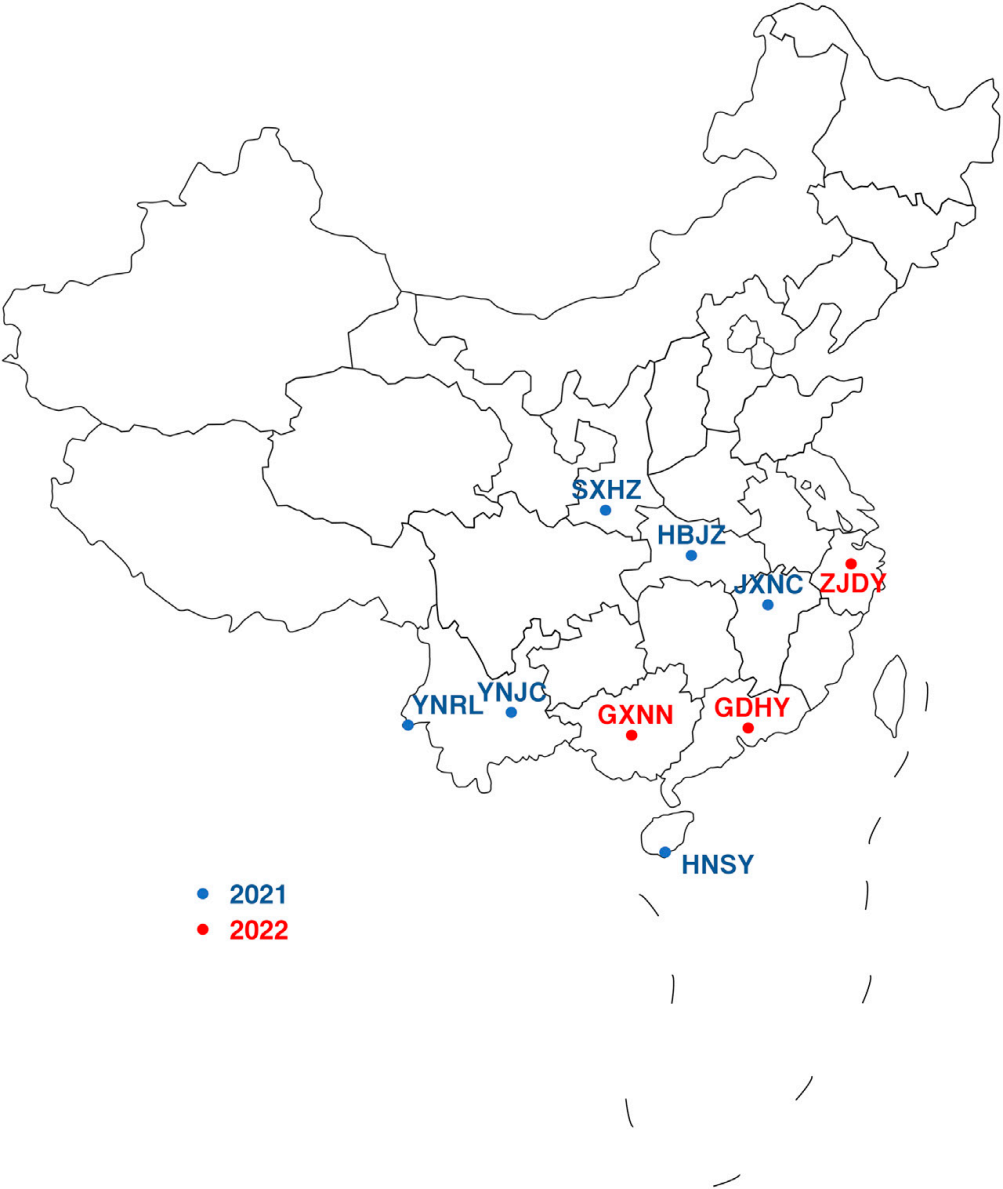
Sampling locations of *Spodoptera frugiperda* field populations from China.

The field populations of *S. frugiperda* were collected from field crops (primarily maize) that were grown in Ruili of Yunnan (RL), Jiangcheng of Yunnan (JC), Sanya of Hainan (SY), Jingzhou of Hubei (JZ), Hanzhong of Shanxi (HZ), Nanchang of Jiangxi (NC), Dongyang of Zhejiang (DY), Huiyang of Guangdong (HY) and Nanning of Guangxi (NN) during 2021–2022 (Figure 1 and Supplementary Table S1). A susceptible strain (SS) of *S. frugiperda* originated from a population in Yunnan maize fields in 2019 and was reared on an artificial diet without any insecticide exposure in the laboratory. Third-instar larvae of the parental (F_0_) or first generation (F_1_) were used for the susceptibility bioassay. *S. frugiperda* populations were then reared under chamber conditions [25°C ± 1°C, 70%–80% relative humidity (RH), 14-h/10-h light/dark photoperiod] for several discrete generations until they were used in bioassays.

### 2.2 Reagents

The chlorpyrifos (97.5%), bifenthrin (97%), deltamethrin (98%), lambda-cyhalothrin (97%), phoxim (96%), chlorantraniliprole (95%) and chlorfenapyr (96%) used in this study were technical grade and were provided by Prof. Chaobin Xue (Shandong Agricultural University, Tai’an, China). Triton X-100 was purchased from Sigma‒Aldrich (St. Louis, MO, United States) and acetone (reagent grade) was purchased from Sinopharm Chemical Reagent Co., Ltd.

### 2.3 Diet-overlay bioassay

The ingestion bioassay employed a diet overlay method as described previously (Bolzan et al., 2019). The insecticides were dissolved in acetone as stock solutions and then diluted to a series of concentration gradients (concentration selection based on pre-experiments with different populations) with distilled water containing 0.1% Triton X-100. An aliquot of 40 μL insecticide solution was applied to each well, and after drying, the larvae were placed on contaminated feed (one 3rd instar larva per well), and a solution containing only distilled water and surfactant was used as the control treatment. For complete bioassays, five concentrations, plus a control (without insecticide), were used for each population and a total of 48 larvae were treated for each concentration. Each concentration was replicated three times with sixteen larvae per replicate. All treatments were maintained at 25°C ± 1°C under 70%–80% relative humidity and a 14-h/10-h light/dark photoperiod. Mortality was assessed after 72 h, and larvae showing no coordinated movement were recorded as dead when touched with a brush.

### 2.4 Glass vial bioassay

The concentration-mortality bioassay was administered by a glass vial method as described previously (Mao et al., 2021). The internal surface area of each glass vial (Guangzhou Li-ge Technology Co., LTD.) was 35.2 cm^2^ (diameter was 2.3 cm, and height was 4.3 cm). Concentrations of the insecticide were diluted in acetone, and five-seven concentrations were used to obtain concentration-mortality curves for the SS strain. Briefly, a glass vial containing 350 μL of insecticide solution and a roller mixing device was used to ensure that its interior was evenly coated with insecticide, and a total of thirty 3rd instar larvae were treated for each concentration. There were three replicates for each concentration and 6–9 doses for each insecticide. Mortality was recorded after 60 min of exposure to chlorpyrifos, bifenthrin, deltamethrin, lambda-cyhalothrin and phoxim and after 120 min of exposure to chlorantraniliprole and chlorfenapyr. Glass vials were placed in a chamber at 25°C ± 1°C and 70%–80% RH. For mortality detection in nine field-collected populations, the experimental design was completely randomized with eight replicates per population (10 larval replicates^−1^).

### 2.5 Development and accuracy verification of diagnostic kit

The diagnostic kit was designed to include seven insecticides test vials (chlorpyrifos, bifenthrin, deltamethrin, lambda-cyhalothrin, phoxim, chlorantraniliprole, and chlorfenapyr), and the LD_90_ for each insecticide was used as the discriminating dose based on the susceptible strain of *S. frugiperda* prepared by the glass vial bioassay.

A total of nine field populations were used to test and validate the accuracy of the kit. Mortalities of *S. frugiperda* field populations (RL, JC, SY, JZ, HZ, and NC) were exposed to LD_90_ of seven insecticides determined with the glass vial method and resistance determined with the diet-overlay bioassay. Seven mortality-resistance ratio linear correlation equations were conducted. By detecting the mortalities under the discriminating dose, the theoretical resistance ratio was calculated according to the mortality-resistance ratio correlation equation. Significant difference and correlation analyses between theoretical and actual resistance ratios were performed for seven insecticides evaluated in the DY, HY, and NN field populations.

### 2.6 Assessment of the control failure likelihood

The minimum recommended label rates of registered insecticides are shown in Supplementary Table S2 (http://www.chinapesticide.org.cn/). Based on the toxicity regression equation, the minimum recommended concentration of insecticide is converted to obtain the achieved mortality, and the achieved mortality is calculated with the expected mortality (80%) according to the following formula to evaluate the control failure likelihood. The control failure likelihood (CFL) = [1- achieved mortality (%)/expected mortality (e.g., 80%)] × 100%, where the value of the control failure likelihood >0% indicates a risk of control failure (Guedes, 2017).

### 2.7 Statistical analysis

The mortality data were corrected using the control mortality with Abbott’s formula (Abbott, 1925). Probit analysis was performed to evaluate the LC_50_ (the insecticide concentration that is required to kill 50% of the tested larvae) value, LC_90_ (the insecticide concentration that is required to kill 90% of the tested larvae) value, confidence limit (95% CL) and slopes, χ^2^ and degrees of freedom (*df*) (Finney, 1964). Mortality of *S. frugiperda* to insecticides in different field populations was tested by one-way ANOVA and Tukey’s multiple comparison test, and differences with different small letters were statistically significant (*p* < 0.05). The correlation between mortality and the resistance ratio was calculated using Pearson’s method, as well as significant differences between theoretical and actual resistance ratios using *t*-tests via IBM SPSS Statistics 20.0, and *p* values that were less than 0.05 were considered statistically significant.

## 3 Results

### 3.1 Development of a diagnostic kit to detect insecticide resistance

To rapidly detect *S. frugiperda*’s resistance levels to insecticides, a diagnostic time of 1–2 h was determined by pretesting based on a susceptible strain. The LD_90_ (0.98, 0.84, 0.38, 1.64, 0.0082, 1.75 and 0.65 μg/cm^2^, respectively) of chlorpyrifos, bifenthrin, deltamethrin, lambda-cyhalothrin, phoxim, chlorantraniliprole and chlorfenapyr was identified as the discriminating dose (Table 1). Under this dose, the mortality of the tested insecticide against the SS strain ranged from 88.8% to 95% (Table 2), which was in line with the expected mortality of 90%.

**TABLE 1:** The toxicity of seven insecticides to the susceptible strain (SS) of *S. frugiperda* determined with the glass vial method.

Insecticide	LD_50_/(μg/cm^2^)^a^	95% confidence limit/(μg/cm^2^)	LD_90_/(μg/cm^2^)	95% confidence limit/(μg/cm^2^)	Slope (SE)	χ^2^ (*df*)
chlorpyrifos	0.25	0.20 ∼ 0.33	0.98	0.69 ∼ 1.64	2.19 (0.27)	2.78 (4)
bifenthrin	0.20	0.15 ∼ 0.26	0.84	0.62 ∼ 1.26	2.05 (0.23)	0.90 (5)
deltamethrin	0.10	0.061 ∼ 0.14	0.38	0.29 ∼ 0.51	2.24 (0.33)	1.03 (4)
lambda-cyhalothrin	0.49	0.39 ∼ 0.61	1.64	1.22 ∼ 2.48	2.43 (0.27)	2.72 (5)
phoxim	0.0017	0.0011 ∼ 0.0024	0.0082	0.0060 ∼ 0.012	1.89 (0.21)	2.82 (5)
chlorantraniliprole	0.43	0.33 ∼ 0.55	1.75	1.24 ∼2.94	2.10 (0.27)	0.57 (4)
chlorfenapyr	0.25	0.21 ∼ 0.30	0.65	0.50 ∼ 0.96	3.08 (0.38)	3.67 (4)

^a^ Values represent the practical insecticide dose (μg/cm2) in each glass tube = the insecticide concentration (ng/μL) × 350 (μL)/inner surface area of the glass vial (cm2).

**TABLE 2:** Mortalities of *S. frugiperda* field populations exposed to LD_90_ of seven insecticides determined with the glass vial method and resistance determined with the diet-overlay bioassay.

Insecticide	Population	Mortality under discriminating dose^a^ of insecticide/%	LC_50_ (95% confidence limit) mg/L	Resistance ratio^b^
chlorpyrifos	SS	95.0 ± 1.89 ab	39.4 (29.5 ∼ 61.4)	1.00
	RL	32.5 ± 6.74 d	246 (224 ∼ 280)	6.24
	JC	92.5 ± 2.50 abc	47.3 (36.3∼ 65.8)	1.20
	SY	96.3 ± 1.83 a	38.2 (30.6 ∼ 49.7)	0.97
	JZ	80.0 ± 3.27 c	82.4 (68.7∼ 106)	2.09
	HZ	97.4 ± 1.83 a	18.9 (14.2 ∼ 27.0)	0.48
	NC	81.3 ± 2.95 bc	68.6 (55.5 ∼ 91.9)	1.74
bifenthrin	SS	92.5 ± 1.64 a	6.92 (5.47 ∼ 8.89)	1.00
	RL	88.8 ± 1.25 a	14.2 (11.0 ∼ 17.9)	2.05
	JC	85.0 ± 3.27 a	12.5 (9.89 ∼ 15.8)	1.80
	SY	41.3 ± 4.41 c	151 (103 ∼ 274)	21.8
	JZ	70.0 ± 2.67 b	52.6 (43.8 ∼ 65.6)	7.60
	HZ	93.8 ± 2.63 a	6.12 (3.93 ∼ 11.9)	0.88
	NC	67.5 ± 4.91 b	66.9 (53.3 ∼ 93.2)	9.66
deltamethrin	SS	95.0 ± 1.89 a	9.57 (7.50 ∼ 12.6)	1.00
	RL	90.0 ± 2.50 a	12.3 (9.43 ∼ 15.4)	1.28
	JC	92.5 ± 1.89 a	9.05 (7.44 ∼ 11.1)	0.95
	SY	75.0 ± 1.89 b	133 (95.5 ∼ 223)	13.9
	JZ	83.8 ± 2.63 ab	92.0 (83.4 ∼ 105)	9.61
	HZ	90.0 ± 4.23 a	10.1 (8.09 ∼ 13.0)	1.06
	NC	86.3 ± 3.24 ab	75.6 (60.1 ∼ 104)	7.90
lambda-cyhalothrin	SS	90.0 ± 1.89 a	15.4 (12.7 ∼ 18.9)	1.00
	RL	60.0 ± 3.27 cd	144 (117 ∼ 177)	9.34
	JC	73.8 ± 3.24 bc	74.6 (59.7 ∼ 89.4)	4.84
	SY	77.5 ± 3.66 ab	44.1 (37.6 ∼ 52.7)	2.86
	JZ	73.8 ± 3.24 bc	89.6 (75.4 ∼ 111)	5.81
	HZ	57.5 ± 3.66 d	138 (115 ∼ 176)	8.98
	NC	78.8 ± 2.95 ab	50.5 (41.3 ∼ 63.3)	3.28
phoxim	SS	88.8 ± 1.25 a	1.13 (1.01 ∼ 1.34)	1.00
	RL	31.3 ± 2.95 e	22.0 (19.8 ∼ 24.9)	19.5
	JC	51.3 ± 2.95 c	18.6 (16.5 ∼ 20.9)	16.5
	SY	70.0 ± 2.67 b	3.78 (3.29 ∼ 4.42)	3.35
	JZ	55.0 ± 2.23 c	10.2 (8.45∼ 12.5)	9.00
	HZ	37.5 ± 3.66 de	21.6 (18.1 ∼ 28.8)	19.2
	NC	48.8 ± 3.50 cd	13.1 (10.8∼17.5)	11.6
chlorantraniliprole	SS	90.0 ± 1.89 ab	0.65 (0.51 ∼ 0.82)	1.00
	RL	88.8 ± 2.27 ab	0.45 (0.33 ∼ 0.62)	0.69
	JC	53.8 ± 3.24 cd	1.58 (1.29 ∼ 2.02)	2.43
	SY	81.3 ± 3.50 b	0.90 (0.69 ∼ 1.31)	1.38
	JZ	45.0 ± 3.78 d	1.68 (1.30 ∼ 2.16)	2.58
	HZ	96.3 ± 1.83 a	0.13 (0.10∼ 0.15)	0.20
	NC	63.8 ± 3.75 c	1.38 (1.20 ∼ 1.63)	2.12
chlorfenapyr	SS	90.0 ± 1.89 a	2.19 (1.77 ∼ 2.69)	1.00
	RL	75.0 ± 3.78 b	3.90 (3.22 ∼ 4.76)	1.78
	JC	72.5 ± 3.66 b	5.21 (4.02 ∼ 7.29)	2.38
	SY	55.0 ± 3.27 c	9.24 (7.74 ∼ 11.2)	4.22
	JZ	22.5 ± 2.50 e	13.1 (11.3∼ 15.6)	5.98
	HZ	73.8 ± 1.83 b	6.94 (5.70 ∼ 9.44)	3.15
	NC	38.8 ± 4.79 d	12.0 (10.2 ∼ 14.2)	5.48

^a^ The LD_90_ of each insecticide against the susceptible strain of *S. frugiperda* by the glass vial method was identified as the discriminating dose.

^b^ Resistance ratio (RR) = LC_50_ value of field population/LC_50_ value of susceptible strain.

### 3.2 Detection by the kit in field populations of *S. frugiperda*

Pests that were collected from six field populations (RL, JC, SY, JZ, HZ and NC) were used to rapidly determine their susceptibility to seven insecticides by using the diagnostic kit. As shown in Table 2, all six *S. frugiperda* strains exhibited resistance to lambda-cyhalothrin, phoxim and chlorfenapyr relative to the SS strain, with diagnostic mortality ranging from 55.7% to 78.8%, 31.3%–70.0% and 22.5%–75.0%, respectively. Only one field population remained susceptible to bifenthrin and chlorantraniliprole (mortality ≥90%), while the other five populations showed resistance to bifenthrin (41.3%–88.8%) and chlorantraniliprole (45.0%–88.8%). In addition, half of the field populations remained susceptible to chlorpyrifos at the diagnostic dose. The detection results showed that the kit could quickly distinguish between resistant and sensitive populations and assessed their susceptibility levels to the seven insecticides within 1–2 h.

### 3.3 Verification of kit accuracy

**FIGURE 2:**
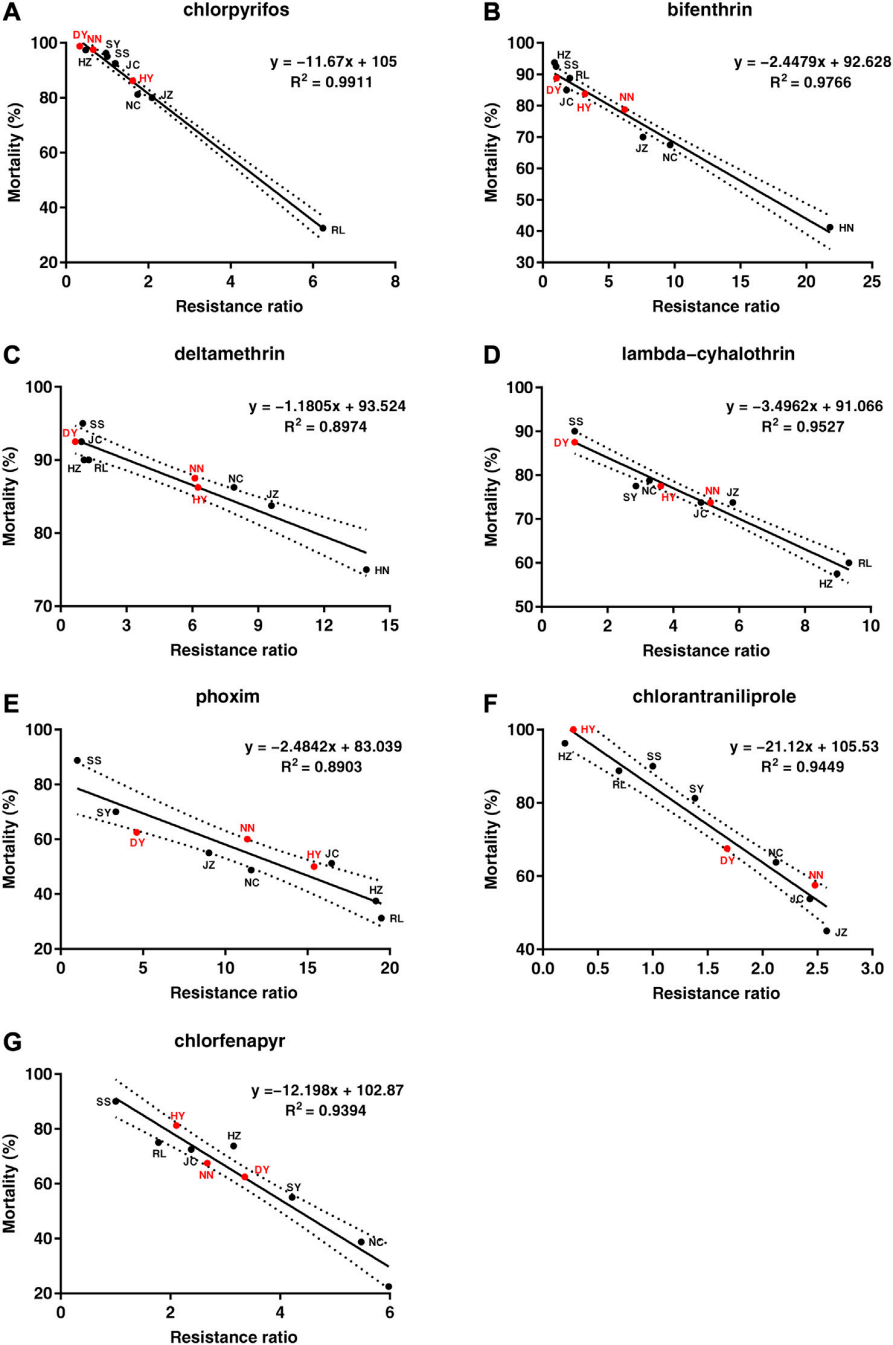
The correlation analysis between the mortality of *Spodoptera frugiperda* field populations under the discriminating dose of seven insecticides and the resistance ratio. **(A)** chlorpyrifos, **(B)** bifenthrin, **(C)** deltamethrin, **(D)** lambda-cyhalothrin, **(E)** phoxim, **(F)** chlorantraniliprole, **(G)** chlorfenapyr. The black spot represents the mortality-resistance ratio for the SS, RL, JC, SY, JZ, HZ and NC field populations. The red spot represents the mortality-resistance ratio for the three field populations (DY, HY and NN). The dashed line represents the 95% confidence interval for the mortality-resistance ratio linear equation.

The LC_50_ values that were obtained from the diet-overlay bioassay were 18.9–246 mg/L (chlorpyrifos), 6.12–151 mg/L (bifenthrin), 9.05–133 mg/L (deltamethrin), 44.1–144 mg/L (lambda-cyhalothrin), 3.78–22.0 mg/L (phoxim), 0.13–1.68 mg/L (chlorantraniliprole) and 3.90–13.1 mg/L (chlorfenapyr) for the six field populations (Table 2). By combining the mortalities at diagnostic doses with the insecticide resistance ratios as measured by the diet-overlay bioassay, seven mortality-resistance ratio linear correlation equations were obtained (Figure 2). Furthermore, a pairwise correlation analysis showed that the diagnostic mortality of the field population was significantly correlated with the resistance ratio for chlorpyrifos (*r* = −0.996, *p* = 0.000), bifenthrin (*r* = −0.988, *p* = 0.000), deltamethrin (*r* = −0.947, *p* = 0.001), lambda-cyhalothrin (*r* = −0.976, *p* = 0.000), phoxim (*r* = −0.944, *p* = 0.001), chlorantraniliprole (*r* = −0.972, *p* = 0.000) and chlorfenapyr (*r* = −0.969, *p* = 0.000) (Table 3).

**TABLE 3:** Pairwise correlation coefficient comparison between the mortalities of *S. frugiperda* field populations under the discriminating doses of seven insecticides and the resistance ratio.

	Resistance ratio						
	Chlorpyrifos	Bifenthrin	Deltamethrin	Lambda-cyhalothrin	Phoxim	Chlorantraniliprole	Chlorfenapyr
Mortality	−0.996** (*p* = 0.000)	−0.988** (*p* = 0.000)	−0.947** (*p* = 0.001)	−0.976** (*p* = 0.000)	−0.944** (*p* = 0.001)	−0.972** (*p* = 0.000)	−0.969** (*p* = 0.000)

**Negative correlation between mortality and the resistance ratio at the 0.01 level.

Additionally, three populations (DY, HY and NN) of *S. frugiperda* that were collected in the field in 2022 were used to verify the accuracy of the kit. Exposure to the diagnostic doses of the seven insecticides that exhibited mortality corresponded to field populations of DY (98.8%, 95.0%, 93.8%, 91.3%, 62.5%, 67.5% and 62.5%), HY (86.3%, 83.8%, 86.3%, 77.5%, 50.0%, 100% and 81.3%) and NN (97.5%, 78.8%, 87.5%, 73.8%, 60.0%, 57.5% and 67.5%) (Table 4). The theoretical resistance ratios that were calculated from the mortality-resistance ratio correlation equation ranged from 0.54 to 8.27, 0.26–13.3 and 0.64–9.27 for DY, HY and NN, respectively, all of which were within the 95% confidence interval of the linear equation, while the resistance ratios that were determined by the diet-overlay bioassay were 0.33–4.62, 0.028–15.4 and 0.66–11.3 (Table 4), with significant correlations between the two data sets (*r* = 0.960, *p* = 0.000), and no significant differences (*p* > 0.05) were found between the theoretical and actual resistance ratios in the three field populations (Table 4).

**TABLE 4:** Verification of the accuracy of the tested resistance levels to seven insecticides in the field populations of *S. frugiperda* under the discriminating dose.

Insecticide	Correlation equation	Population	Mortality under the diagnostic dose/%	Theoretical resistance ratio	Actual resistance ratio^a^	*p*-value^b^
chlorpyrifos	y = −11.7x + 105	DY	98.8	0.54	0.33	0.098
		HY	86.3	1.61	1.62	0.940
		NN	97.5	0.64	0.66	0.896
bifenthrin	y = −2.45x + 92.6	DY	95.0	1.58	1.04	0.672
		HY	83.8	3.63	3.20	0.795
		NN	78.8	5.67	6.21	0.844
deltamethrin	y = −1.33x + 95.2	DY	93.8	0.87	0.66	0.490
		HY	86.3	6.16	6.26	0.819
		NN	87.5	5.10	6.11	0.896
lambda-cyhalothrin	y = −3.50x + 91.1	DY	91.3	1.02	1.00	0.969
		HY	77.5	3.88	3.62	0.843
		NN	73.8	4.95	5.13	0.890
phoxim	y = −2.48x + 83.0	DY	62.5	8.27	4.62	0.253
		HY	50.0	13.3	15.4	0.454
		NN	60.0	9.27	11.3	0.428
chlorantraniliprole	y = −21.1x + 106	DY	67.5	1.80	1.68	0.580
		HY	98.8	0.32	0.28	0.511
		NN	57.5	2.27	2.48	0.170
chlorfenapyr	y = −12.2x + 103	DY	62.5	3.31	3.36	0.928
		HY	81.3	1.77	2.11	0.464
		NN	67.5	2.90	2.67	0.665

^a^ Actual resistance ratio = LC_50_ value of field population/LC_50_ value of susceptible strain.

^b^ *p*-value of less than 0.05 was thought to be statistically significant.

### 3.4 Assessment of the control failure likelihood by the diagnostic kit

**FIGURE 3:**
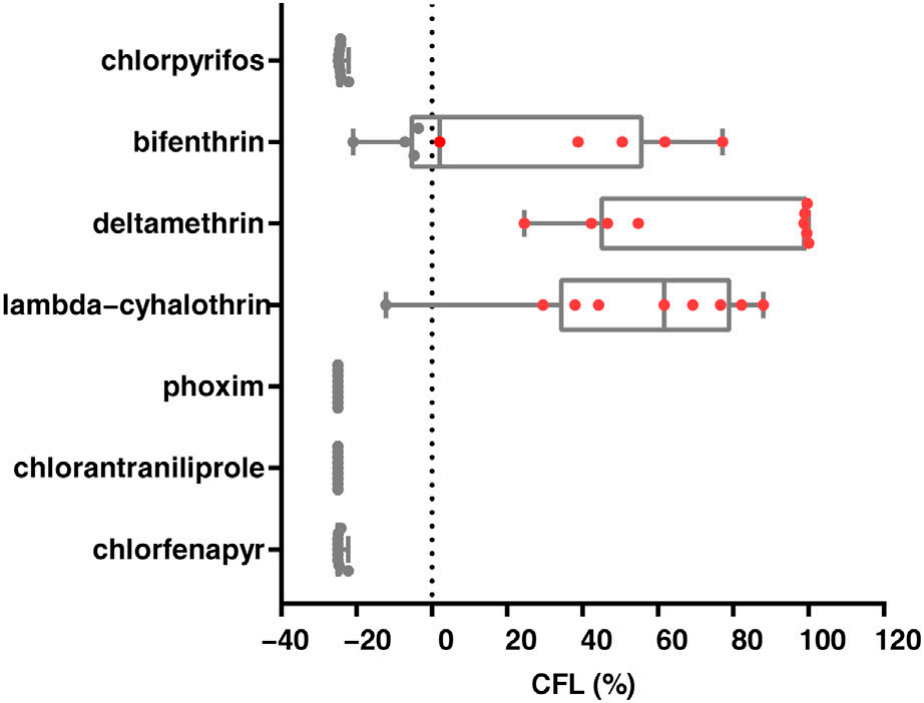
Likelihood of control failure of seven insecticides for *Spodoptera frugiperda* in nine field populations. Each dot represents a population, while the red dots (CFL> 0%) represent populations that control failure likelihood.

We conducted studies that estimated the likelihood of chemical control failure in nine field populations of *S. frugiperda*. The likelihood of control failure for deltamethrin was reported in all populations, and lambda-cyhalothrin and bifenthrin were at risk of control failure in one and five out of nine populations, respectively. Potential control failure populations were not identified for chlorpyrifos, phoxim, chlorantraniliprole and chlorfenapyr (Figure 3). Furthermore, a significant negative correlation was found between diagnostic mortality and the likelihood of control failure for bifenthrin (*r* = −0.899, *p* = 0.001), deltamethrin (*r* = −0.737, *p* = 0.024) and lambda-cyhalothrin (*r* = −0.871, *p* = 0.002) (Supplementary Table S3). Finally, three linear models were constructed for bifenthrin (y = −1.99x + 175), deltamethrin (y = −4.25x + 443) and lambda-cyhalothrin (y = −2.98x + 271), and based on this, it was hypothesized that the diagnostic mortality rate could only be used to control brown flies after exceeding 88.4%, 100% and 91.2%, respectively.

## 4 Discussion

Resistance monitoring and recording are critical to understanding and addressing both existing and developing pesticide resistance issues, and the development of reliable bioassays facilitates the reporting, sharing and straightforward comparison of resistance data (Chanda et al., 2016; Sparks et al., 2021). Here, we present a diagnostic kit that enables practitioners and farmers to quickly detect the resistance levels of *S. frugiperda* to seven insecticides and thereby reduce the impact of *S. frugiperda* on crop yields worldwide.

The kit was developed based on a glass vial assay, which is suitable for diagnosing susceptibility to a wide range of insecticide classes, thus meeting the criteria for easier use (Snodgrass, 1996). The bioassays in this study (30 min vial drying time and health assessments at 1 or 2 h) were designed to maximize efficiency, which is much easier than the diet-overlay bioassay or leaf disc method (Lira et al., 2020; Chen and Palli, 2022). In addition, the low cost of monitoring ensures the sustainability of enacted plans (Sikaala et al., 2014). In previous studies, we developed rapid diagnostic kits for resistance to seven insecticides based on the approach in *S. furcifera*, and the simple glass vials greatly reduced monitoring costs (Mao et al., 2021). Glass vials that contain a given insecticide can be prepared in advance and can be made in large quantities with simple roller equipment. Having an easy method with inexpensive equipment increases its likelihood to be adopted by crop scouts, extension agents, and growers to conduct resistance testing. Given the advantages of the kit’s low cost and simplicity of operation, it is possible to increase the number of sentinel sites for *S. frugiperda* on a national scale, such as community-based monitoring (CDCP, 2010; Sikaala et al., 2014), which allows the continuous detection of resistance changes during the annual in and out migration periods (Ge et al., 2022).

This diagnostic kit has greater efficiency (1 or 2 h diagnosis time) and is easier to implement than the traditional bioassay method. As a comparison, all kit-based assays can be performed immediately after field collection of *S. frugiperda*, whereas other bioassays usually complete testing at F1 or F2, which results in a minimum of 1 month to obtain resistance data (Jin et al., 2021b; Li et al., 2022). Because the *S. frugiperda* has overlapping generations in the field, it is guaranteed that insects of the appropriate life stage required for the test will be present. This not only minimizes the risk of not detecting resistant populations due to loss of selection pressure when rearing multiple generations in the laboratory (Tabashnik et al., 1994; Zhang et al., 2021), but also allows for a “race” with the pest, as the feeding of *S. frugiperda* is phenomenal. Once this kit is promoted at the agroecosystem level, farmers are expected to screen available insecticides in a short time, thus avoiding the use of ineffective crop protection compounds and the loss of valuable products from the overuse of single insecticides.

Using a diagnostic dose method can provide rapid and reliable monitoring to support the widespread implementation of resistance monitoring. The WHO has established criteria that indicate resistance in mosquitoes if they have a mortality that is less than 90% to insecticides (WHO, 2013). In the present study, the LD_90_ values of the insecticides that were identified for susceptible *S. frugiperda* were used as the diagnostic doses, and they were able to effectively separate susceptible and resistant populations of *S. frugiperda* from nine geographical populations. Furthermore, linear equations for the mortality-resistance ratio were obtained for seven insecticides that were based on six field populations and susceptible strains. Subsequently, the validity of this equation was verified in the other three field populations. The high correlation indicated that the actual resistance ratios that were estimated by the linear equation were consistent with those obtained with the diet-overlay bioassay. In particular, we found a high risk of control failure for the control of *S. frugiperda* with bifenthrin, deltamethrin and lambda-cyhalothrin (Figure 3), and these results are consistent with those of previous studies also showing that invasive *S. frugiperda* populations have developed resistance to pyrethroids (Li et al., 2022), which points to the relevance of assessing the likelihood of control failure based on the concentration of the pesticide in commercial formulations. Notably, we found a significant negative correlation between diagnostic mortality and the likelihood of control failure (bifenthrin, deltamethrin and lambda-cyhalothrin), and based on the model set, the detection criteria that would need to be met for the insecticide to be reintroduced. In addition, we found that chlorpyrifos, phoxim, chlorantraniliprole and chlorfenapyr had good control effects on *S. frugiperda* (CFL < −22%), which indicates that the use of these insecticides can continue to be recommended, but the risk of cross-resistance needs to be added to resistance management (Zhao et al., 2020). The results provide important guidance on the value of re-excavating insecticides.

The development of timely and accurate resistance monitoring methods in resistance monitoring and management must be integrated into *S. frugiperda* control programs so that available insecticides can be used judiciously and the efficacy of chemical-based control can be sustained for the long term (Mao et al., 2019b; Tay et al., 2023). The standardization of testing techniques and the promotion of products can facilitate extensive cooperation between agriculture technical extension centers or local plant protection stations, family farms and individual households so that the effectiveness of resistance management can be maximized (Chanda et al., 2016). The successful development of this kit demonstrates that this methodology can be applied to other lepidopteran pests, as well as to different insecticides.

## Data Availability

The original contributions presented in the study are included in the article/Supplementary Material, further inquiries can be directed to the corresponding author.
