# Supplementary material for: Rapid test to detect insecticide resistance in field populations of Spodoptera frugiperda (Lepidoptera: Noctuidae)
Source: Front Physiol. 2023 Aug 23;14:1254765. doi: 10.3389/fphys.2023.1254765 (PMC10482100; doi:10.3389/fphys.2023.1254765)
Supplement: Supplementary file 1 [file Table1.DOCX]

**Table S1. Sample sites, collection dates and developmental stages of *Spodoptera frugiperda* collected from fields in China**

| Locations | Site | Collection Date | Host plant | Developmental stage | Larvae number |
| --- | --- | --- | --- | --- | --- |
| Ruili, Yunnan | 97.82°E, 24.01°N | Mar. 2021 | Maize | 2rd - 6th instars | 117 |
| Jiangcheng,Yunnan | 101.87°E, 22.59°N | Apr. 2021 | Maize | 3rd - 6th instars | 96 |
| Sanya, Hainan | 109.12°E, 18.37°N | Mar. 2021 | Maize | 2rd - 6th instars | 323 |
| Jingzhou, Hubei | 112.58°E, 30.22°N | Jul. 2021 | Maize | 2rd - 6th instars | 232 |
| Hanzhong, Shanxi | 116.68°E, 40.12°N | Jul. 2021 | Maize | 2rd - 5th instars | 186 |
| Nanchang, Jiangxi | 115.96°E, 28.55°N | Jul. 2021 | Maize | 2rd - 6th instars | 75 |
| Dongyang, Zhejiang | 120.32°E, 29.29°N | Aug. 2022 | Broomcorn | 4rd - 6th instars | 93 |
| Huiyang, Guangdong | 114.30°E, 22.95°N | Sep. 2022 | Maize | 3rd - 6th instars | 118 |
| Nanning, Guangxi | 108.28°E, 23.16°N | Sep. 2022 | Maize | 2th - 6th instars | 152 |
