# Supplementary material for: Rapid test to detect insecticide resistance in field populations of Spodoptera frugiperda (Lepidoptera: Noctuidae)
Source: Front Physiol. 2023 Aug 23;14:1254765. doi: 10.3389/fphys.2023.1254765 (PMC10482100; doi:10.3389/fphys.2023.1254765)
Supplement: Supplementary file 2 [file Table2.DOCX]

**Table S2. Minimum recommended dose of registered insecticides to** ***S. frugiperda***

| Insecticide | Minimum recommended dose (mg/L) | Formulation^a^ (Active ingredient content) | Origin |
| --- | --- | --- | --- |
| chlorpyrifos | 667 | EC (40%) | Zhejiang Xinnong Chemical Co., Ltd |
| bifenthrin | 41.7 | ME (2.5%) | ADAMA Huifeng (Jiangsu) Co., Ltd |
| deltamethrin | 8.33 | EC (25 g/L) | Bayer Co., Ltd |
| lambda-cyhalothrin | 50.0 | EW (10%) | ADAMA Huifeng (Jiangsu) Co., Ltd |
| phoxim | 1000 | EC (40%) | Nanjing Huazhou Pharmaceutical Co., Ltd |
| chlorantraniliprole | 80.0 | SC (200 g/L) | FMC Co., Ltd |
| chlorfenapyr | 110 | SC (10%) | BASF SE |

^a^ SC (suspension concentrate), EW (emulsion in Water), ME (microemulsion), EC (emulsifiable concentrate).
