# Supplementary material for: Rapid test to detect insecticide resistance in field populations of Spodoptera frugiperda (Lepidoptera: Noctuidae)
Source: Front Physiol. 2023 Aug 23;14:1254765. doi: 10.3389/fphys.2023.1254765 (PMC10482100; doi:10.3389/fphys.2023.1254765)
Supplement: Supplementary file 3 [file Table3.DOCX]

**Table S3. Pairwise correlation coefficient comparison between the mortalities of *S. frugiperda* field populations under the discriminating doses of three insecticides and the control failure likelihood**

|  | Control failure likelihood | | |
| --- | --- | --- | --- |
|  | bifenthrin | deltamethrin | lambda-cyhalothrin |
| Mortality | −0.899**  (*P* = 0.001) | −0.737*  (*P* = 0.024) | −0.871**  (*P* = 0.002) |

* Negative correlation between the mortality and the resistance ratio at the 0.05 level.

** Negative correlation between the mortality and the resistance ratio at the 0.01 level.
